# Washed microbiota transplantation improves clinical symptoms, gut microbiota, and metabolic profiles in autism spectrum disorder in a twin cohort

**DOI:** 10.3389/fmicb.2026.1885281

**Published:** 2026-07-17

**Authors:** Shuo Feng, Xinyu Si, Caimei Lu, Zheng Gao, Jiangyan Wang, Qingqing Yang, Shenghua Lu, Ting Su, Juan Yang, Xingxiang He, Lei Wu

**Affiliations:** 1 Department of Gastroenterology and Microbiota Medicine, The First Affiliated Hospital of Guangdong Pharmaceutical University, Guangzhou, China; 2 Guangdong Provincial Key Laboratory for Research and Evaluation of Pharmaceutical Preparations, Guangzhou, China

**Keywords:** autism spectrum disorder, metabolomics, metagenomics, microbiota–gut–brain axis, twin study, washed microbiota transplantation

## Abstract

**Objective:**

Autism spectrum disorder (ASD) is a heterogeneous neurodevelopmental condition characterized by impaired social communication, repetitive behaviors, and restricted interests. Dysregulation of the microbiota–gut–brain axis is closely associated with the pathogenesis of ASD. Washed microbiota transplantation (WMT) has emerged as a promising intervention for ASD, but existing cohort studies lack genetically identical controls, making it difficult to distinguish intervention-related changes from genetic and environmental confounding factors. This twin-paired controlled study adopted a study design that minimizes the influence of genetics and shared environment, to explore the associations of WMT with clinical symptoms, gut microbiota, and metabolic profiles in children with ASD.

**Methods:**

Three pairs of age- and environment-matched twins (one ASD-affected, one typically developing sibling) were enrolled. WMT was administered to the ASD participant in each pair. Fecal samples were collected at baseline and post-intervention. Gut microbiota and metabolic profiles were analyzed using metagenomic sequencing and targeted metabolomics, respectively. Clinical outcomes were evaluated using the Childhood Autism Rating Scale (CARS), Autism Behavior Checklist (ABC), Sleep Disturbance Scale for Children (SDSC), and Bristol Stool Form Scale (BSFS). Relevant observations were carried out to explore potential changing trends.

**Results:**

After WMT, CARS, ABC, SDSC, and BSFS exhibited small numerical directional shifts toward healthier values, but none reached statistical significance. Gut microbial structure and function presented a shifting trend toward the profile of their typically developing twin siblings. Abnormal lipid and energy metabolism indicators showed partial ameliorative trends, and the number of differential metabolites between ASD patients and healthy siblings was markedly reduced. Tyrosine and phenylalanine metabolic pathways, together with *Segatella*, *Negativibacillus*, and *Sangeribacter*, may be associated with incomplete phenotypic changes in this cohort.

**Limitations:**

Although the twin-pair design has high internal validity and can provide strong causal inference evidence for the effect of microbiota transplantation in treating ASD, this study has limitations such as a small sample size, a single-center non-randomized observational design. All findings in this pilot study are merely descriptive trends, and the relevant mechanism analysis only provides correlational clues. A single session of microbiota transplantation failed to fully adjust aromatic amino acid metabolism in ASD children. No definitive causal relationship can be concluded based on the findings of this small-sample pilot study.

**Conclusion:**

Under tightly controlled genetic and environmental conditions, gut microbial dysbiosis presents correlational characteristics with ASD-related phenotypes. WMT was associated with consistent remodeling of gut microbial ecology and partial resolution of metabolic dysregulation in ASD children, with multi-omic signatures converging toward healthy twins. Clinical rating scales only displayed non-significant minor numerical shifts, which cannot be interpreted as evidence of clinical symptom improvement. These initial findings provide exploratory mechanistic clues and phenotypic data supporting WMT as a targeted microbiome intervention approach for ASD, and await further validation through large-scale randomized controlled trials.

**Clinical trial registration:**

Identifier ChiCTR2400091105.

## 1 Introduction

Autism spectrum disorder (ASD) is a neurodevelopmental disorder defined by persistent deficits in social communication and interaction, as well as restricted, repetitive patterns of behavior, interests, or activities (American Psychiatric Association, 2022). The global prevalence of ASD has risen continuously in recent years, imposing a substantial disease burden on affected families and public health systems. According to the latest epidemiological data, the prevalence of ASD among 8-year-old children in developed countries has reached 1/36, and the prevalence in some regions of China is also close to 1%, and it is still on the rise (Feng et al., 2025). Although the cause of ASD has not been fully clarified, existing evidence indicates that its onset is a complex result of the combined effects of genetic susceptibility, intestinal microecology (Fulceri et al., 2016; Strati et al., 2017; Chaidez et al., 2014), immune inflammation (Fung et al., 2017; Powell et al., 2017; Sharon et al., 2016; Sharon et al., 2019; Tomova et al., 2020), nutritional metabolism (Nogay and Nahikian-Nelms, 2019), and environmental exposure (Li et al., 2019), among others. Dysfunction of the microbiota–gut–brain axis (MGBA) is considered a key pathway linking peripheral disturbances to central nervous system developmental abnormalities.

Nevertheless, the causal direction of the gut microbiota–ASD association remains actively debated. An alternative perspective posits that observed microbial alterations may largely represent secondary consequences of ASD-associated traits — including highly prevalent food selectivity, restricted dietary patterns, chronic gastrointestinal symptoms, and concurrent medication use — rather than primary drivers of neurodevelopmental pathophysiology (Sharon et al., 2019; Wu et al., 2025; Mitchell et al., 2026; Zhang et al., 2025). Resolving this question is critical for establishing whether microbiota-targeted interventions can exert clinically meaningful effects on ASD phenotypes. Twin models, which largely eliminate genetic and shared environmental noise, therefore represent a powerful approach to resolve this controversy and clarify whether microbial alterations correlate with ASD phenotypes independent of dietary and behavioral confounders.

A growing body of clinical and preclinical evidence highlights the critical role of the MGBA in ASD pathogenesis. Children with ASD frequently exhibit reduced gut microbial diversity, enrichment of potentially pathogenic taxa, and depletion of beneficial commensals (Feng et al., 2025). These alterations are accompanied by disturbances in microbial metabolites including short-chain fatty acids, bile acids, and amino acid derivatives (Strati et al., 2017; Hsiao et al., 2013). Through metabolic, immune, neural, and endocrine pathways, the gut microbiota modulates neurodevelopment, synaptic plasticity, microglial activation, and inflammatory tone, thereby directly or indirectly influencing ASD-related behaviors (Powell et al., 2017; Sharon et al., 2019; Tomova et al., 2020).

Washed microbiota transplantation (WMT) is an optimized form of fecal microbiota transplantation (FMT) featuring standardized washing, purification, and concentration steps. Different from conventional manual FMT that only conducts simple homogenization and rough filtration, WMT adopts automated and standardized multi-round centrifugation, impurity removal and concentration procedures. It effectively removes fecal debris, food residues, parasite eggs and pro-inflammatory metabolites retained in conventional FMT preparations, while retaining high-activity commensal microbiota. This technical optimization significantly enhances clinical safety and microbial engraftment efficiency, and realizes full-process operational standardization which facilitates multi-center clinical replication. Multiple studies and the Nanjing Consensus on Washed Microbiota Transplantation have confirmed that the repeated washing and centrifugation steps of WMT can effectively remove fecal debris, residual food residues, viruses and pro-inflammatory metabolites (leukotriene B4, prostaglandin G2, etc.) that are retained in conventional FMT preparations. Clinical data show that the overall adverse event rate of WMT is significantly lower than that of manual FMT, and the risks of abdominal pain, transient diarrhea and inflammatory reactions after transplantation are greatly reduced, which is particularly suitable for vulnerable populations such as children with ASD (Feng et al., 2025; Zhang et al., 2020; Shi, 2020; Zhang et al., 2026). WMT has shown therapeutic potential in gastrointestinal disorders, metabolic syndrome, and neurological conditions (Wu et al., 2022b; Hu et al., 2024; Lin et al., 2025; Wu et al., 2022a; Wu et al., 2023; Lin et al., 2024; Liang et al., 2024; He et al., 2024; Zhang et al., 2020). Preliminary clinical studies suggest that WMT may alleviate gastrointestinal dysfunction, behavioral abnormalities, sleep disturbances, and social deficits in children with ASD. However, most existing studies are single-arm, small-scale, and lack rigorous controls. None have used genetically matched controls to eliminate confounding from heredity and household environment, and making it difficult to accurately disentangle the true intervention effect of WMT from confounding factors (Feng et al., 2025).

Twins represent an ideal model to control for genetic and shared environmental variation. By using a twin-paired design, this study aimed to determine the specific effects of WMT on clinical symptoms, gut microbial structure and function, and fecal metabolic profiles in children with ASD. Through multi-omics integration and correlation analysis, it provides preliminary observational data under matched genetic backgrounds, so as to accumulate clues for further exploring the characteristics and application of WMT in ASD.

## 2 Materials and methods

### 2.1 Study design and ethics approval

This single-center, prospective, twin-paired controlled study was conducted at the First Affiliated Hospital of Guangdong Pharmaceutical University (Guangzhou, China). The study protocol was reviewed and approved by the Ethics Committee of the First Affiliated Hospital of Guangdong Pharmaceutical University (Approval No. 2024-IIT-39). This ethical approval covers a series of independent sub-studies investigating WMT in neurodevelopmental disorders, including the present twin-paired ASD study. All datasets, participants, clinical interventions, omics analyses, and endpoints in this manuscript are exclusive and independent from any previously published work.

Legal guardians provided written informed consent prior to enrollment. The study was conducted in accordance with the Declaration of Helsinki and ethical guidelines for clinical research involving human participants.

### 2.2 Study participants

Participants were enrolled between June 2019 and October 2025.

Inclusion criteria: Three pairs of twins were included, with one member of each pair meeting the DSM-5 (American Psychiatric Association, 2013) ASD diagnostic criteria and the other having no ASD or other neuro-psychiatric disorders, and their age, living environment were matched.

Exclusion criteria: Non-twin status or both twins diagnosed with ASD; Severe gastrointestinal diseases, immunodeficiency, or severe hepatic/renal dysfunction; History of allergic reaction to WMT donor materials; Antibiotic use within 1 month prior to WMT; Major systemic diseases or other conditions deemed incompatible with the study.

During the study period, all participants were instructed to maintain their usual diet and daily routines. Any changes in medication, diet, or concurrent therapies were monitored and recorded throughout the study period. Parents/guardians were interviewed at each visit to confirm adherence to these protocols and to document any deviations.

### 2.3 WMT administration

Compared with conventional fecal microbiota transplantation, the whole preparation process of WMT in this study is fully automated and standardized. Conventional manual FMT lacks unified operation specifications and only relies on simple filtration, leading to inconsistent quality of bacterial suspension across batches. In contrast, our WMT protocol implements strict parameter control for homogenization, filtration, centrifugation and concentration to ensure the stability of finished products. WMT was performed using a standardized automated purification system (GenFMTer, Nanjing FMT Medical Technology Co., Ltd.). Donor screening and preparation of washed microbiota suspension followed previously published protocols (Pan et al., 2022; Zhong et al., 2023).

#### 2.3.1 Donor screening criteria and health monitoring

##### 2.3.1.1 Comprehensive past medical history assessment

All donors were recruited from the standardized donor bank of the Guangdong Provincial Engineering Technology Research Center for Microbiota Therapy. During pre-enrollment, we collected: Lifetime medical history, including any past gastrointestinal, infectious, neuropsychiatric, or autoimmune diseases; 12-month medication history; Lifestyle factors (diet, exercise, smoking, alcohol consumption); 6-month travel history; Family history of genetic, metabolic, or neuropsychiatric disorders; Donors with any conditions that could affect gut microbiota function were excluded.

##### 2.3.1.2 Real-time monitoring during donation

All donors underwent daily health checks before each donation: Standardized questionnaire for acute symptoms (fever, diarrhea, abdominal pain, respiratory symptoms); Body temperature measurement; 24-h medication use review; Donors were temporarily suspended if they reported any acute illness, took medications, or had changes in bowel habits until full recovery was achieved. Conduct a comprehensive re-examination (including blood and stool tests) of active donors every 3 months.

All donors enrolled in this study were healthy adults from standardized donor banks. Considering the current global shortage of high-quality age-matched pediatric donor resources, we adopted strictly screened adult donors for intervention. Our research team will carry out follow-up comparative trials between adult donors and pediatric donors to further optimize donor selection strategies for children with ASD.

#### 2.3.2 WMT preparation

Preparation steps: Fresh fecal samples (50-100 g) were collected from donors and processed within 1 h. Homogenization with sterile saline (1:5 w/v). After being filtered and processed by an intelligent separation system, solid particles, fungi and parasite eggs and other impurities are removed. Three cycles of centrifugation (700 g, 3 min, 4 °C) and resuspension in sterile saline. Final suspension adjusted to 10^13^ CFU/mL concentration. Administration: Before WMT treatment, all the children underwent transendoscopic enteral tubing (TET) under colonoscopy. The tip of the catheter was placed at the ileocecal region of the colon, and the external end was fixed to the left buttock. The fresh prepared bacterial solution was slowly infused through the TET tube. The infusion was carried out for 6 consecutive days, with a daily dose of 60–90 mL. Pre- and post-procedure care: During the treatment period, the child was instructed to maintain a light diet and avoid strenuous exercise. After each infusion of the bacterial liquid, patients remained in the right lateral position for 2 h. Before treatment and avoided antibiotics for 1 month before and after intervention.

### 2.4 Clinical assessment

Clinical evaluations were performed at baseline and after completion of WMT intervention.

CARS (Childhood Autism Rating Scale): 15-item scale assessing communication, social interaction, emotional response, and behavioral patterns (Schopler et al., 1980).

ABC (Autism Behavior Checklist): 58-item scale covering sensory, social, motor, language, and self-care domains (Brinkley et al., 2007).

SDSC (Sleep Disturbance Scale for Children): Evaluated sleep quality and disturbances (Bruni et al., 1996).

BSFS (Bristol Stool Form Scale): Assessed stool consistency and gastrointestinal function (Lewis and Heaton, 1997).

The clinical reviewers who completed the CARS, ABC, SDSC and BSFS evaluations were trained professional clinicians. All reviewers maintained an objective attitude towards the intervention time points (baseline and post-intervention), compared the post-treatment conditions with those from the previous assessment, and communicated with the parents of the patients to confirm the situation, in order to eliminate subjective assessment biases.

### 2.5 Sample collection and processing

Fresh fecal samples were collected within 10 min of defecation by guardians and stored immediately at −80 °C until analysis. Samples were collected at baseline and post-intervention from ASD patients and at baseline from healthy co-twins.

### 2.6 Metagenomic sequencing

Total metagenomic DNA was extracted from 200–250 mg frozen human stool (wet weight) using the DNeasy PowerFecal Pro DNA Kit (Qiagen, Hilden, Germany) following manufacturer’s protocols with standardized modifications at Novogene Co., Ltd. Briefly, stool homogenates mixed with lysis beads were subjected to bead-beating on TissueLyser II at 30 Hz for three cycles of 30 s with 30 s intervals, followed by 10 min incubation at 70 °C to enhance cell lysis and inhibitor removal. RNase A was supplemented to eliminate RNA contamination prior to DNA purification. Two wash steps were extended to 2 min room-temperature incubation to reduce humic acid and bile salt impurities, and DNA was eluted in 50 μL nuclease-free water with 3 min static incubation to increase final concentration. For stool samples with high human host DNA proportion, NEBNext Microbiome DNA Enrichment Kit was applied to deplete methylated host genomic fragments. Extracted DNA underwent multi-stage quality control: double-stranded DNA concentration was quantified via Qubit 3.0 Fluorometer; OD₂₆₀/₂₈₀ and OD₂₆₀/₂₃₀ ratios were measured by NanoDrop One spectrophotometer, with qualified thresholds set as 1.80–2.00 and ≥1.70, respectively. DNA integrity was assessed using 1% agarose gel electrophoresis, and high-molecular-weight DNA for long-read metagenomics was further validated by Agilent 2,100 Bioanalyzer. Only DNA meeting total amount ≥400 ng and concentration >24 ng/μL was retained for subsequent library construction and shotgun metagenomic sequencing. Quality control was performed using Agilent 5,400 and qPCR. Sequencing was performed on the Illumina NovaSeq platform (PE150). Raw reads were quality-filtered, and host contamination was removed using Bowtie2 (Chen et al., 2018; Karlsson et al., 2012). *De novo* assembly was performed using MEGAHIT (Li et al., 2015). Open reading frames were predicted and quantified (Qin et al., 2010). Taxonomic and functional annotations were performed against KEGG, eggNOG, CAZy, VFDB, PHI, and CARD databases. Bioinformatic analyses including *α*/*β*-diversity, LEfSe, and ANOSIM were performed in R (Huson et al., 2011; McArthur et al., 2013).

### 2.7 Targeted metabolomics

Fecal metabolites were extracted and analyzed using UHPLC–MS/MS (ExionLC AD UHPLC-QTRAP 6500+, AB SCIEX). Separation used a Waters HSS T3 column (2.1 × 150 mm) at 40 °C. Mobile phases consisted of 0.1% formic acid (A) and acetonitrile/isopropanol (1:1, B). Multivariate statistical analyses including PCA, OPLS-DA, and differential metabolite screening (VIP > 1.0, |FC| > 1.2, *p* < 0.05) were performed. Metabolic pathway enrichment was conducted using the KEGG database (Heischmann et al., 2016; Haspel et al., 2014; Sreekumar et al., 2009).

### 2.8 Statistical analysis

Continuous data were expressed as mean ± standard deviation. Within-group comparisons (pre- vs. post-intervention) used paired Wilcoxon signed-rank test. Between-group comparisons used independent t-test or Mann–Whitney U test. Correlations were analyzed using Spearman correlation coefficient. A *p* < 0.05 was considered statistically significant. All analyses were performed using R software.

Notably, given the extremely small sample size (3 twin pairs) of this pilot study, Spearman correlation analysis was only used for exploratory observation of data trends. Correlation results in this study are for descriptive reference only and cannot support definitive correlation or regulatory inferences.

Notably, given the extremely small sample size (3 twin pairs) of this pilot study, formal correction for multiple comparisons (e.g., FDR adjustment) was not applied, as such corrections would be overly conservative at this sample size and could obscure meaningful exploratory patterns. All statistical results should be interpreted as descriptive and hypothesis-generating rather than confirmatory. Standardized notation throughout the Results section: all adjusted values are uniformly written as FDR-adjusted *p*, and unadjusted *p*-values from clinical and metabolomic analyses are uniformly written as *p*, to avoid any ambiguity.

## 3 Results

### 3.1 Clinical characteristics

A total of 3 pairs of twins were included, with an average age of 6.0 ± 2.65. The gender ratio of ASD patients was 3:0 (male:female), while the gender ratio of normal twins was 1:2 (male:female). The baseline clinical characteristics of children with ASD are summarized in Table 1.

**Table 1:** Baseline conditions of ASD patients.

Indicator name	Baseline
CARS	37.5 ± 4.44 (*n =* 3)
Interpersonal communication	2.83 ± 0.29 (*n =* 3)
Imitation (words and actions)	2.83 ± 0.29 (*n =* 3)
Emotional response	2.33 ± 0.29 (*n =* 3)
Physical manipulation ability	2.67 ± 0.29 (*n =* 3)
Relationship with non-living objects	2.5 ± 0.5 (*n =* 3)
Adaptation to environmental changes	2.0 ± 0.5 (*n =* 3)
Visual response	2.5 ± 0.5 (*n =* 3)
Auditory response	2.67 ± 0.58 (*n =* 3)
Near-sense response	2.33 ± 0.29 (*n =* 3)
Anxiety response	2.33 ± 0.58 (*n =* 3)
Verbal communication	2.67 ± 0.58 (*n =* 3)
Non-verbal communication	2.33 ± 0.29 (*n =* 3)
Highly active	2.33 ± 0.76 (*n =* 3)
Intellectual function	2.33 ± 0.58 (*n =* 3)
General impression	2.83 ± 0.29 (*n =* 3)
ABC	60.67 ± 20.98 (*n =* 3)
S (Sensory ability)	12.0 ± 4.36 (*n =* 3)
R (Social interaction ability)	8.33 ± 2.52 (*n =* 3)
B (Motor ability)	12.33 ± 10.69 (*n =* 3)
L (Language ability)	17.0 ± 8.19 (*n =* 3)
S (Self-care ability)	11.0 ± 3.0 (*n =* 3)
SDSC	50.0 ± 15.87 (*n =* 3)
BSFS	2.67 ± 0.58 (*n =* 3)
Age	6.0 ± 2.65 (*n =* 3)
BMI	18.40 ± 2.33 (*n =* 3)
White blood cell count (10^9^/L)	7.95 ± 1.41 (*n =* 3)
Lymphocyte count (10^9^/L)	3.77 ± 0.96 (*n =* 3)
Lymphocyte Percentage (%)	0.47 ± 0.042 (*n =* 3)
Neutrophil count (10^9^/L)	3.20 ± 0.64 (*n =* 3)
Platelet count (10^9^/L)	311.7 ± 29.02 (*n =* 3)
Hemoglobin concentration (g/L)	129.3 ± 4.73 (*n =* 3)
Alanine aminotransferase (U/L)	16.33 ± 3.77 (*n =* 3)
Aspartate aminotransferase (U/L)	27.33 ± 5.03 (*n =* 3)
Serum albumin (g/L)	46.20 ± 1.02 (*n =* 3)
Globulin (g/L)	22.40 ± 2.95 (*n =* 3)
Total Bilirubin (μmol/L)	8.20 ± 2.10 (*n =* 3)
Direct bilirubin (μmol/L)	1.97 ± 0.31 (*n =* 3)
Indirect bilirubin (μmol/L)	6.23 ± 2.26 (*n =* 3)
Gastrin-17 (pmol/L)	17.24 ± 23.52 (*n =* 3)

### 3.2 WMT was accompanied by improving trends of clinical manifestations in ASD patients

Descriptive paired analyses were performed for 26 clinical indicators before and after WMT, with data presented as mean ± standard deviation. Within-group comparisons were conducted using the paired Wilcoxon signed-rank test, consistent with the statistical methods specified in Section 2.8. The study was designed as a paired sample (comparison before and after treatment for the same subject), with a sample size of *n =* 3. Descriptive analysis of paired data showed that mean scores of CARS, ABC, SDSC, and BSFS moved in a numerically favorable direction after WMT intervention. However, none of these changes reached statistical significance (all *p* > 0.05) due to the extremely small sample size. These numerical shifts should be interpreted solely as exploratory observational trends and do not constitute evidence of clinical efficacy. The direction of numerical change is consistent with larger open-label WMT cohorts reported by our group and others (Feng et al., 2025; Pan et al., 2022; Zhong et al., 2025; Zheng et al., 2025; Pan et al., 2025; Zhang et al., 2022; Liu et al., 2023; Hu et al., 2025). These preliminary feasibility data may inform sample size estimation for future randomized controlled trials, but no conclusion regarding clinical efficacy can be drawn from this pilot study (Table 2).

**Table 2:** Comparison of clinical data of patients with ASD before and after receiving WMT treatment.

Indicator name	Before treatment	After treatment	*p*-value
CARS	37.5 ± 4.44	35.83 ± 3.75	0.25
Interpersonal communication	2.83 ± 0.29	2.67 ± 0.29	>0.999999
Imitation (words and actions)	2.83 ± 0.29	2.5 ± 0.5	0.5
Emotional response	2.33 ± 0.29	2.17 ± 0.58	>0.999999
Physical manipulation ability	2.67 ± 0.29	2.33 ± 0.76	>0.999999
Relationship with non-living objects	2.5 ± 0.5	2.33 ± 0.58	>0.999999
Adaptation to environmental changes	2.0 ± 0.5	2.0 ± 0.5	
Visual response	2.5 ± 0.5	2.5 ± 0.5	
Auditory response	2.67 ± 0.58	2.5 ± 0.5	>0.999999
Near-sense response	2.33 ± 0.29	2.33 ± 0.29	
Anxiety response	2.33 ± 0.58	2.33 ± 0.58	
Verbal communication	2.67 ± 0.58	2.67 ± 0.58	
Non-verbal communication	2.33 ± 0.29	2.17 ± 0.29	>0.999999
Highly active	2.33 ± 0.76	2.33 ± 0.76	
Intellectual function	2.33 ± 0.58	2.33 ± 0.58	
General impression	2.83 ± 0.29	2.67 ± 0.29	>0.999999
ABC	60.67 ± 20.98	49.0 ± 6.08	0.5
S (Sensory ability)	12.0 ± 4.36	7.33 ± 2.31	0.5
R (Social interaction ability)	8.33 ± 2.52	5.0 ± 1.73	0.25
B (Motor ability)	12.33 ± 10.69	8.67 ± 4.04	0.5
L (Language ability)	17.0 ± 8.19	17.33 ± 6.43	>0.999999
S (Self-care ability)	11.0 ± 3.0	10.67 ± 1.53	>0.999999
SDSC	50.0 ± 15.87	48.33 ± 15.5	0.25
BSFS	2.67 ± 0.58	3.67 ± 0.58	0.5
Age	6.0 ± 2.65	6.0 ± 2.65	

### 3.3 Structural and functional characteristics of the gut microbiota before and after WMT treatment in twin patients with ASD

**Figure 1:**
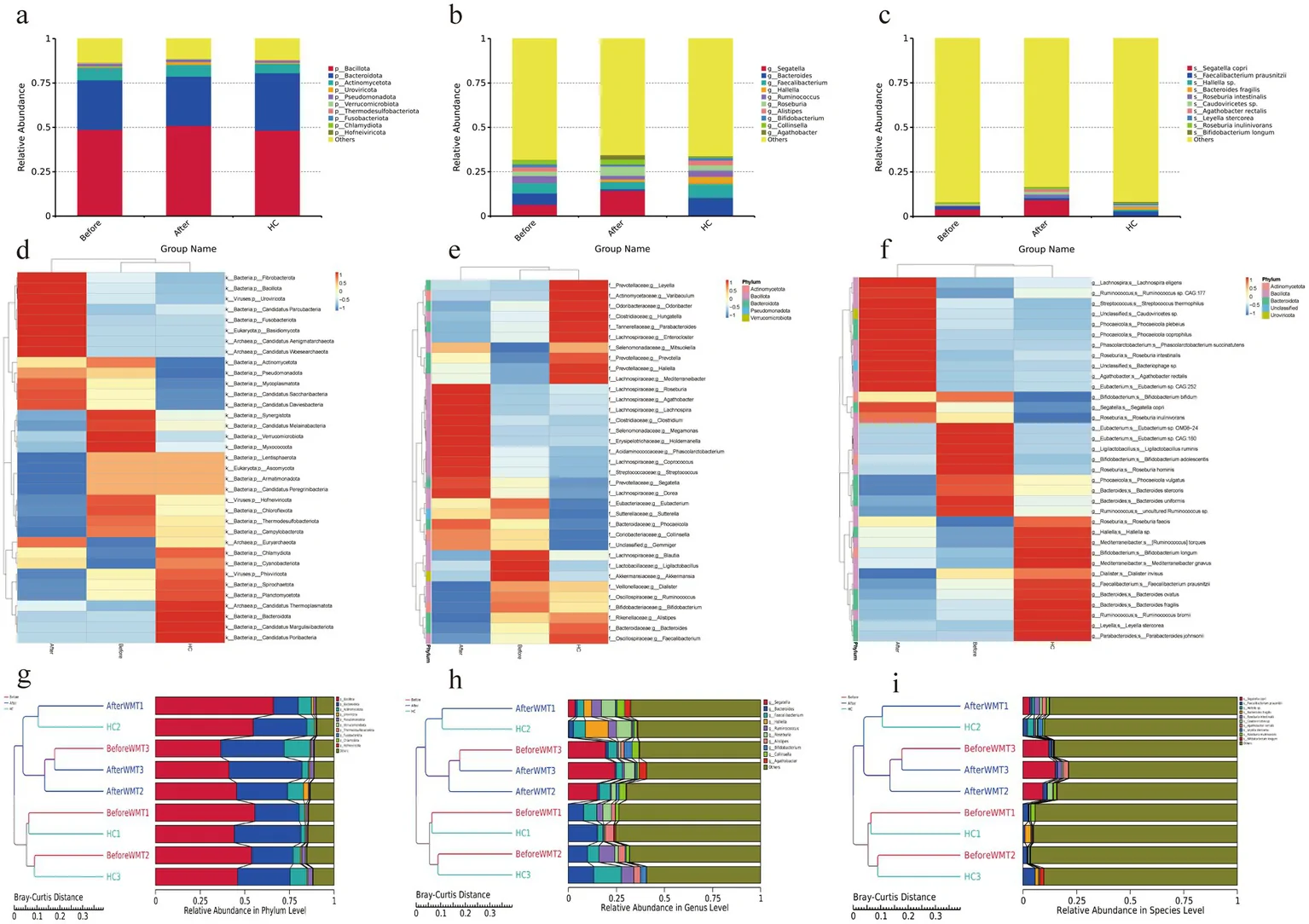
The regulatory effect of WMT on the intestinal microbiota of twins. The composition characteristics of the gut microbiota of the twin subjects **(a,d,g)**, at the genus **(b,e,h)** and species **(c,f,i)** levels; before (Before WMT treatment); After (After WMT treatment); HC (Healthy twins).

We first characterized the overall gut microbial compositional profiles of twin participants. At the phylum level, the intervention mainly affected the fine-tuning of the dominant phyla Bacillota, Bacteroidota and the significant changes in the functional phyla Fusobacteriota, Uroviricota, while the archaeal domain and low-abundance phyla showed no significant response. After WMT treatment, at the genus level, the proportion of *g_Bacteroides* increased, and the proportion of conditionally pathogenic genera such as *g_Escherichia-Shigella* decreased. At the species level, the abundance of species such as *s_Segatella copri* and *s_Bacteroides vulgatus* significantly increased, and the species composition became more diverse, with a higher overlap with the dominant species of the healthy twins. Compared with the healthy twins, there were significant differences in the gut microbiota of autistic patients (Before/After), mainly manifested as imbalance in the proportion of dominant phyla, insufficient abundance of beneficial genera (such as *Faecalibacterium*, *Bifidobacterium*), and abnormal abundance of potential harmful genera (such as *Bacteroides*, *Alistipes*) (Figures 1a–i).

**Figure 2:**
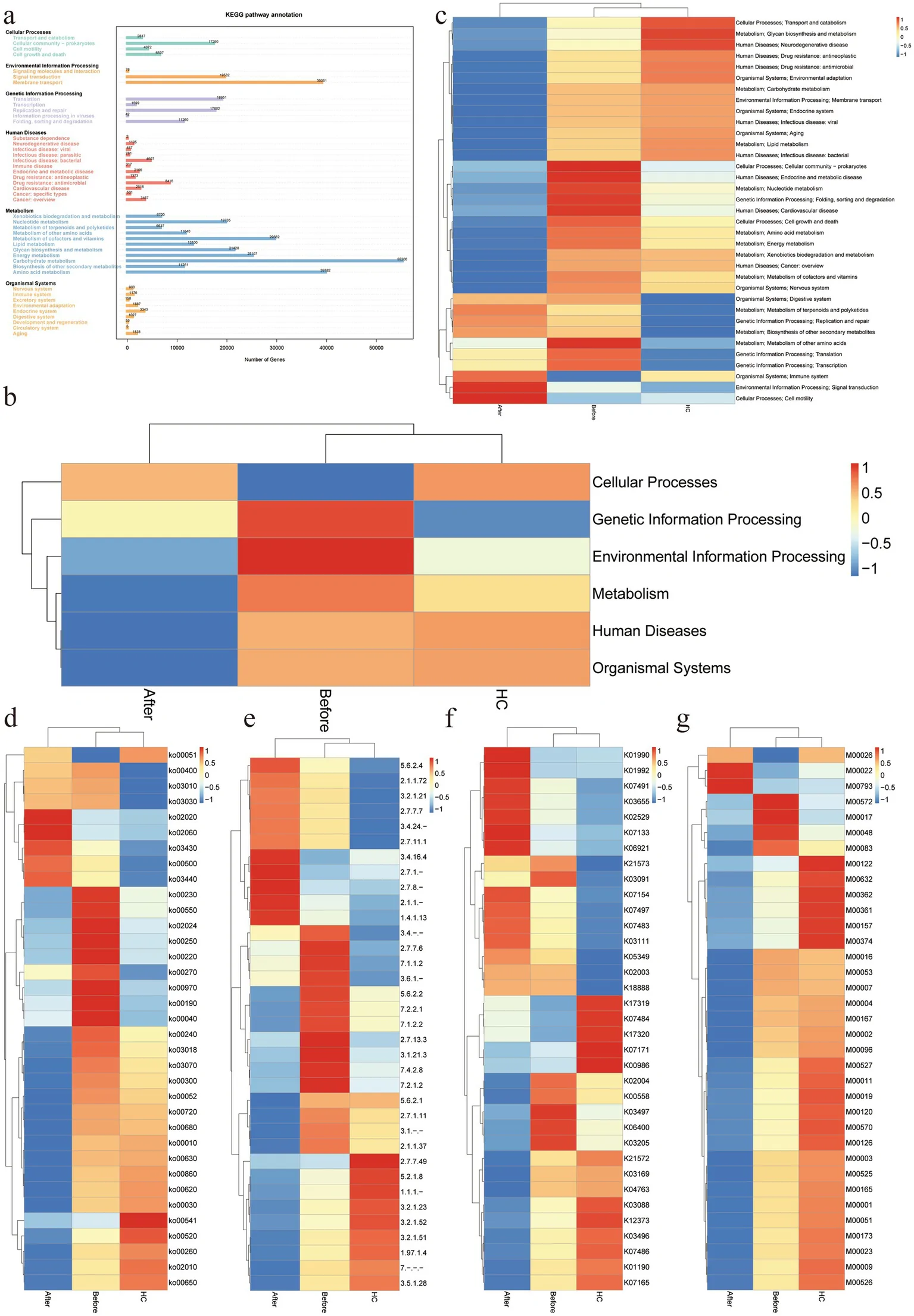
KEGG Functional Abundance clustering heat map: Gene number statistics map **(a)** level1 **(b)**, level2 **(c)**, level3 **(d)**, ec **(e)**, ko **(f)**, module **(g)** levels; before (before WMT treatment); after (after WMT treatment); HC (healthy twins). All abundance changes shown are descriptive trends and did not reach statistical significance.

**Figure 3:**
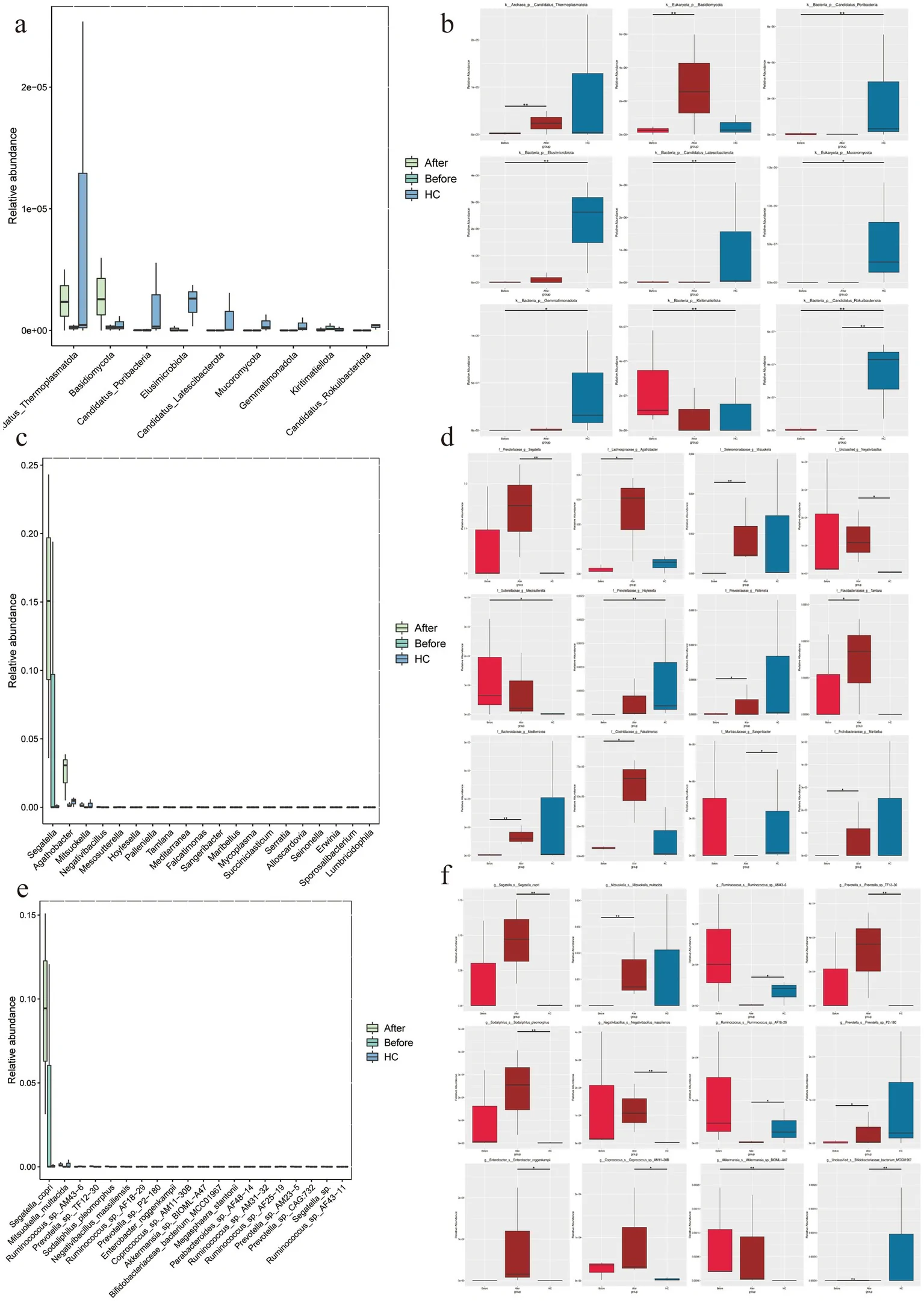
MetaGenomeSeq analysis, phylum **(a,b)**, genus **(c,d)**, and species **(e,f)** levels; before (before WMT treatment); after (after WMT treatment); HC (healthy twins). FDR-corrected *p*.

We next performed differential abundance analysis to identify taxa significantly altered by WMT. At the phylum level, taxa including Portbacteria, Elusimicrobiota, Latescibacterota, Gemmatimonadota, Mucoromycota, and Kiritimatiellota were significantly reshaped after WMT (FDR-adjusted *p* < 0.01 or FDR-adjusted *p* < 0.05), shifting toward the microbiota profile of healthy co-twins. Candidatus, Thermoplasmatota, Basidiomycota showed significant differences after WMT treatment compared to before (FDR-adjusted *p* < 0.01). Candidatus, Rokubacteria showed little difference before and after treatment (Figures 2a,b). *Mesosutterella, Hoylesella* after WMT treatment significantly reshaped the gut microbiota structure of patients with autism (FDR-adjusted *p* < 0.01 or FDR-adjusted *p* < 0.05), making it converge towards the microbiota characteristics of healthy twins. *Agathobacter, Mitsuokella, Paleniella, Mediterranea, Falcrimonas, Maribellus* showed significant differences before and after treatment, but no significant difference compared to healthy twins (FDR-adjusted *p* < 0.01 or FDR-adjusted *p* < 0.05). *Segatella, Negativibacillus, Sangeribacter* showed little difference before and after treatment, but had a significant difference compared to healthy twins and after treatment (FDR-adjusted *p* < 0.01 or FDR-adjusted *p* < 0.05) (Figures 2c,d). At the species level, *Akkermansia* sp. *BIOML-A47* significantly reshaped the gut microbiota structure of patients with ASD after treatment (FDR-adjusted *p* < 0.01), making it converge towards the microbiota characteristics of healthy twins. *Mitsuokella multacida, Prevotella_*sp.*_P2-180* showed significant differences before and after treatment compared to before (FDR-adjusted *p* < 0.01), but no significant difference before and after treatment compared to the healthy twins. *Bifidobacteriaceae_bacterium_MCC01967* showed significant differences before and after treatment, but had a significant difference before and after treatment compared to the healthy twins (FDR-adjusted *p* < 0.01). *Segatella_copri, Prevotella sp. TF12-30, Sodaliphilus pleomorphus, Negativibacillus_massiliensis, Ruminococcus* spp. *(AM43-6, AF18-29), Coprococcus_sp._AM11-30B* showed little difference before and after treatment, but had a significant difference compared to healthy twins and after treatment (FDR-adjusted *p* < 0.01 or FDR-adjusted *p* < 0.05) (Figures 3e,f).

Descriptive analysis of KEGG functional annotation abundance revealed directional shifts in pathway profiles after WMT. None of these changes reached statistical significance, and they are presented as exploratory trends. KEGG functional annotation and abundance analysis. The results showed that before the intervention, the abundances of pathways related to “genetic information processing,” “environmental information processing,” and “human diseases” increased in patients, and after the intervention, they all decreased and approached the levels of healthy twins; the pathological pathways such as “neurodegenerative diseases” and “glycan biosynthesis” were abnormally active before the intervention, and their abundances decreased after the intervention and approached the levels of healthy twins; while the homeostasis pathways such as “cell movement” showed an increase in activity. In addition, core pathological pathways of autism such as the phosphatidylinositol signaling system (ko02020), key enzymes related to oxidative stress and amino acid metabolism, showed high abundance before the intervention, and decreased to the healthy level after the intervention; the core module of glycolysis (M00026) showed insufficient activity before the intervention and recovered to the healthy level after the intervention, while the oxidative phosphorylation module (M00122) showed an opposite repair trend. These results are consistent with the phenotypic characteristics such as elevated oxidative stress and impaired methylation function observed in ASD individuals, and provide descriptive clues for exploring the correlation between WMT and changes of ASD-related functional pathways (Figures 2a–g).

**Figure 4:**
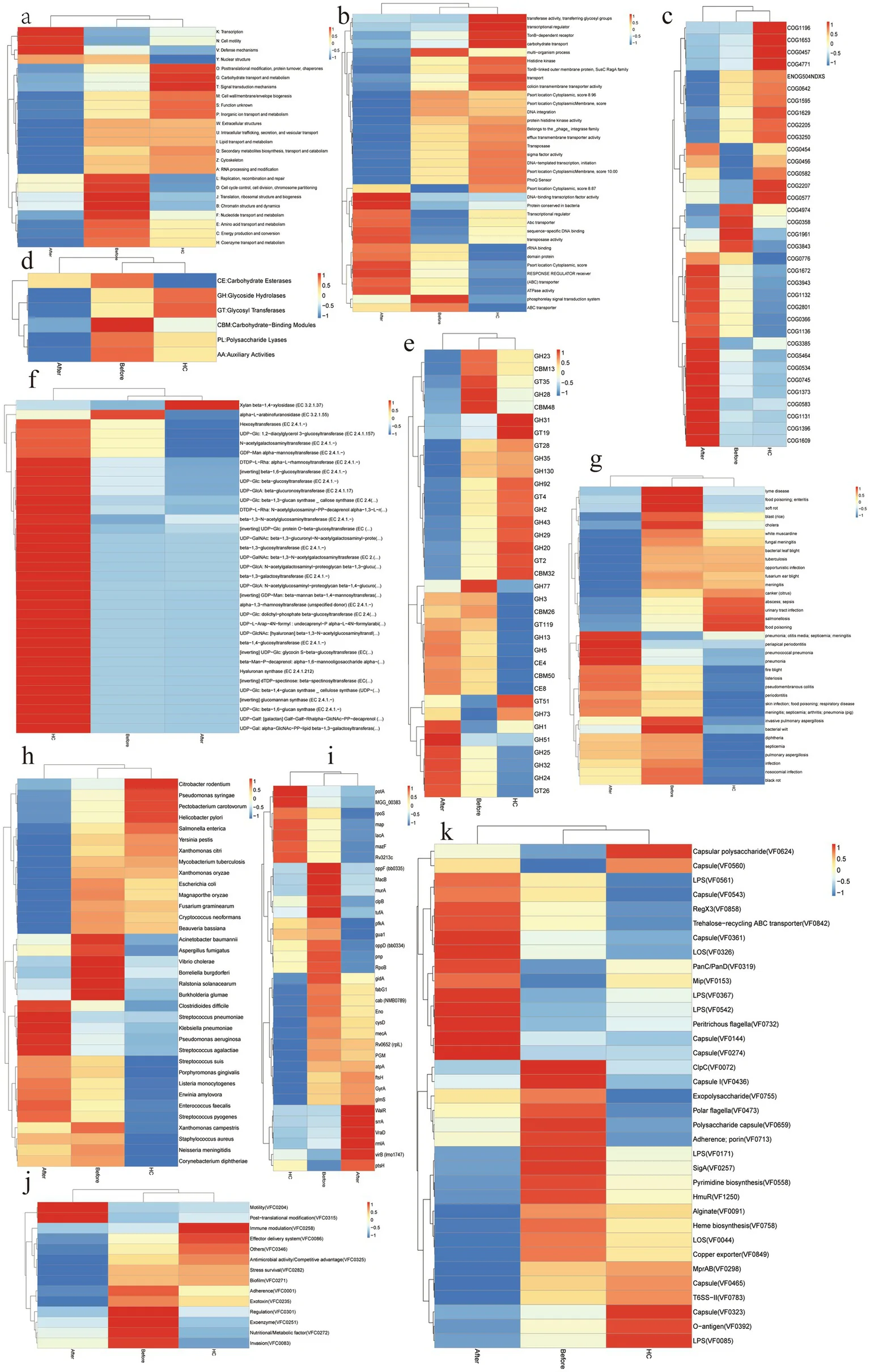
EggNOG functional abundance clustering heatmap: level 1 **(a)**, level 2 **(b)**, og **(c)**; CAZY functional abundance clustering heatmap: level 1 **(d)**, level 2 **(e)**, ec **(f)**; PHI functional abundance clustering heatmap: level 1 **(g)**, level 2 **(h)**, level 3 **(i)**; VEDF functional abundance clustering heatmap: level 1 **(j)**, level 2 **(k)**; before (before WMT treatment); after (after WMT treatment); HC (healthy twins).

Based on the functional annotations of EggNOG, the three levels presented distribution changes related to the intervention, and after the intervention, the proportions of most functional categories, functional descriptions, and direct homologous groups of genes tended to approach those of the healthy twin group. Especially in the transcriptional regulation, defense mechanisms, and the functional distribution of specific homologous group genes, intervention responses were observed, providing important data support for understanding the molecular mechanism of autism intervention (Figures 4a-c). Comparing the different levels of carbohydrate-active enzymes systems in CAZy of patients with autism and healthy individuals, there are inherent differences. This intervention not only did not make the expression patterns of patients approach those of the healthy twin group, but instead expanded the differences between the two. Among them, the CBM module, the GT2/GH2 family, and the corresponding EC elements were the core change targets after the intervention, which can provide molecular basis for the optimization of autism intervention plans and the exploration of intervention mechanisms (Figures 4d-f). Analysis of three levels of PHI database annotations revealed that the disease-related phenotypes, pathogen abundance, and functional gene expression of patients changed selectively before and after the intervention, and there were differences compared with healthy twins (Figures 4g-i). Analysis of VFDB virulence factor annotations revealed that the expression characteristics of virulence factors in patients with autism are closely associated with the disease-related pathological physiological processes. The intervention measures can specifically regulate the expression of key virulence factors to be accompanied by changes of the immune disorders, metabolic imbalance, and abnormal intestinal barrier function in patients, providing important biological basis for understanding the pathogenesis of autism and optimizing intervention strategies (Figures 4j–k).

### 3.4 Metabolic profiling characteristics of twin patients with ASD before and after WMT treatment

**Figure 5:**
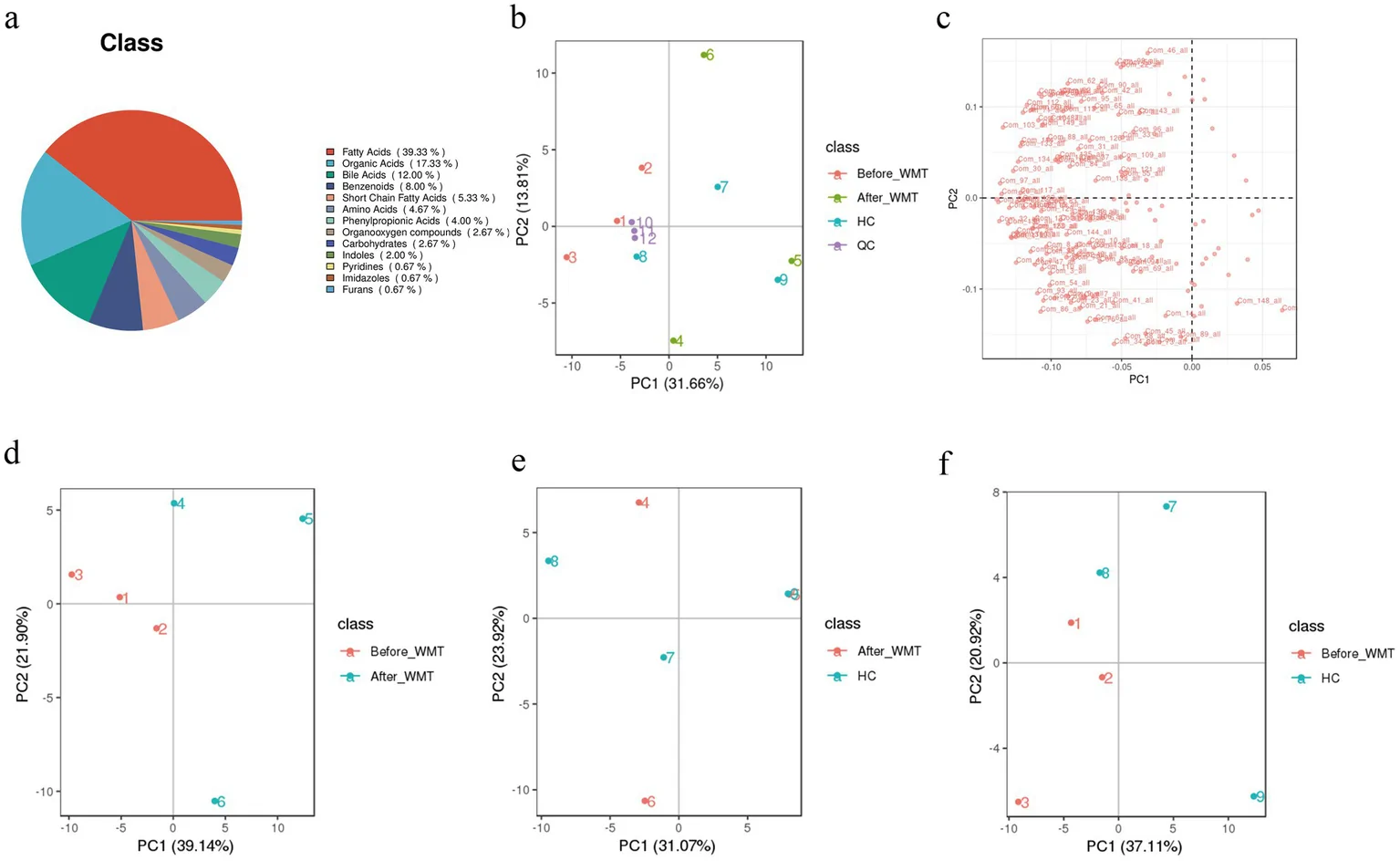
Pie chart of metabolite classification **(a)**; PCA score plots of targeted metabolomics **(b,c)**; Before vs. After **(d)**; After vs. HC **(e)**; Before vs. HC **(f)**; before (before WMT treatment); after (after WMT treatment); HC (healthy twins).

Targeted metabolomics (LC–MS/MS) detected a total of 150 fecal metabolites, mainly including fatty acids (59 types), organic acids (26 types), bile acids (18 types), etc., belonging to 13 categories. The PCA score plot showed a separation trend among the three groups of metabolic profiles (PC1 = 31.66%, PC2 = 13.81%). The metabolic profiles of the autistic patients after WMT treatment were significantly separated from those before the intervention, indicating that WMT intervention was accompanied by notable changes in the metabolic profile, and a subset of metabolic characteristics shifted toward the healthy state after intervention. The intervention effect varies among individuals, and the metabolic characteristics of some patients still show significant differences from those of the healthy twin group (Figures 5a–f).

The criteria for differentiating differences were VIP > 1.0, Fold change > 1.2 or < 0.833, and p < 0.05. Compared with HC, the Before group detected 8 different metabolites, while HC vs. After only detected 2, suggesting that the metabolic profiles of children after WMT were less different from those of healthy siblings (Table 3).

**Table 3:** Statistics of differential metabolites (differential screening: VIP > 1.0, FC > 1.2 or <0.833, *P* < 0.05).

Comparison	Metabolite name	*p*-value	FC	VIP	Mean ± standard deviation
HC vs. Before	L-Lactic acid (down)	0.002394	0.16476	1.62	12.80 ± 0.32 (HC) 15.38 ± 0.47 (Before)
	cis-13,16-Docosadienoic acid (down)	0.027975	0.08654	1.76	5.56 ± 1.22 (HC) 9.03 ± 1.29 (Before)
	trans-10-Heptadecenoic acid (down)	0.030371	0.16928	1.61	5.23 ± 1.07 (HC) 7.95 ± 0.52 (Before)
	Pentadecanoic acid (down)	0.030448	0.19559	1.65	10.01 ± 0.78 (HC) 12.32 ± 0.92 (Before)
	3β -Ursodeoxycholic acid (down)	0.038830	0.14962	1.60	6.81 ± 1.27 (HC) 9.86 ± 0.52 (Before)
	N-Acetyl-L-alanine (down)	0.038971	0.12714	1.67	9.27 ± 1.39 (HC) 12.40 ± 0.98 (Before)
	Isolithocholic acid (down)	0.040002	0.09816	1.73	9.19 ± 1.59 (HC) 12.97 ± 0.62 (Before)
	Hexadecanoic acid (down)	0.046277	0.33894	1.44	14.90 ± 0.62 (HC) 16.43 ± 0.69 (Before)
HC vs. After	Heptanoic acid (up)	0.001691	1.90108	2.86	5.84 ± 0.15 (HC) 4.92 ± 0.10 (After)
	p-Hydroxyphenylacetic acid (down)	0.043923	0.06652	2.23	8.31 ± 1.9 (HC) 12.49 ± 1.56 (After)
Before vs. After	Succinic Acid (up)	0.012554	3.79060	1.15	15.60 ± 0.53 (Before) 13.67 ± 0.56 (After)
	Hyodeoxycholic acid (up)	0.015684	4.28378	1.27	9.09 ± 0.43 (Before) 6.91 ± 0.70 (After)
	N-Acetyl-L-alanine (up)	0.017525	7.80523	1.55	12.40 ± 0.98 (Before) 9.51 ± 0.75 (After)
	Acetylglycine (up)	0.028711	5.82875	1.50	9.97 ± 0.97 (Before) 7.46 ± 0.85 (After)
	cis-13,16-Docosadienoic acid (up)	0.043397	9.94418	1.58	9.03 ± 1.29 (Before) 6.06 ± 0.51 (After)

“Compared Samples: former/latter,” and up-down/down-down indicates that the former is higher/lower in the comparison pair, respectively.

**Figure 6:**
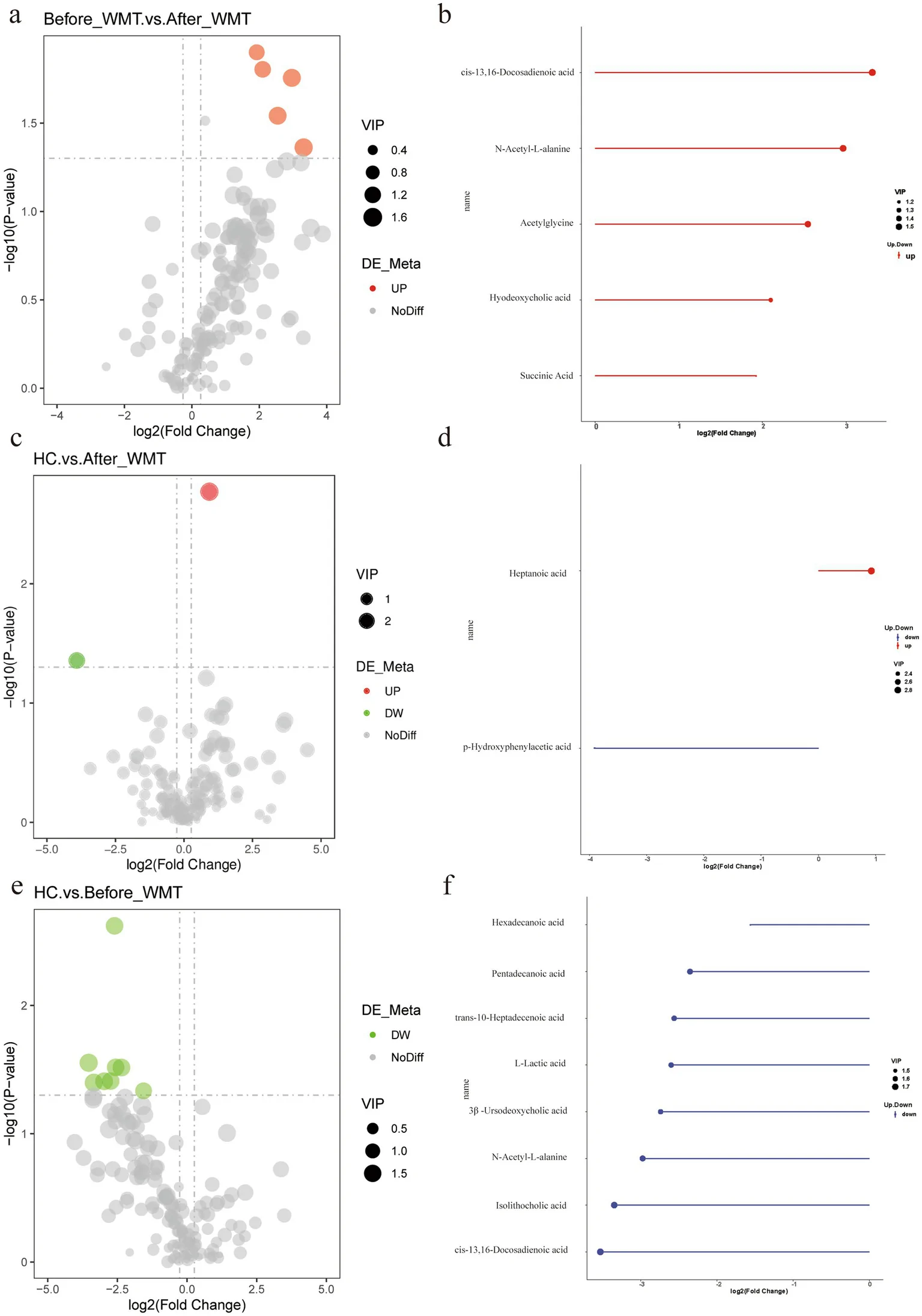
Matchstick diagram and Volcano Map of Differential Metabolites and Overview of differences between groups Before vs. After **(a,b)**, HC vs. After **(c,d)**, HC vs. Before **(e,f)**, before (before WMT treatment); after (after WMT treatment); HC (healthy twins).

In the comparison between the Before and After groups, there were a total of 5 different metabolites that were all upregulated, including Succinic Acid, Hyodeoxycholic acid, N-Acetyl-L-alanine, Acetylglycine, cis-13,16-Docosadienoic acid. In the comparison between the HC and After groups, there were 2 different metabolites that were upregulated -Heptanoic acid and downregulated - p-Hydroxyphenylacetic acid. In the comparison between the HC and Before groups, there were 8 different metabolites that were all downregulated - L-Lactic acid, cis-13,16-Docosadienoic acid, trans-10-Heptadecenoic acid, Pentadecanoic acid, 3β-Ursodeoxycholic acid, N-Acetyl-L-alanine, Isolithocholic acid, Hexadecanoic acid. Compared with HC and After groups, the levels of docosadienoic acid (cis-13,16) and N-acetyl-L-alanine were significantly upregulated in the Before group. There was no overfitting in the fecal metabolic profiles of each group, and significant metabolic differences were observed among the groups (Figures 6a–m).

**Figure 7:**
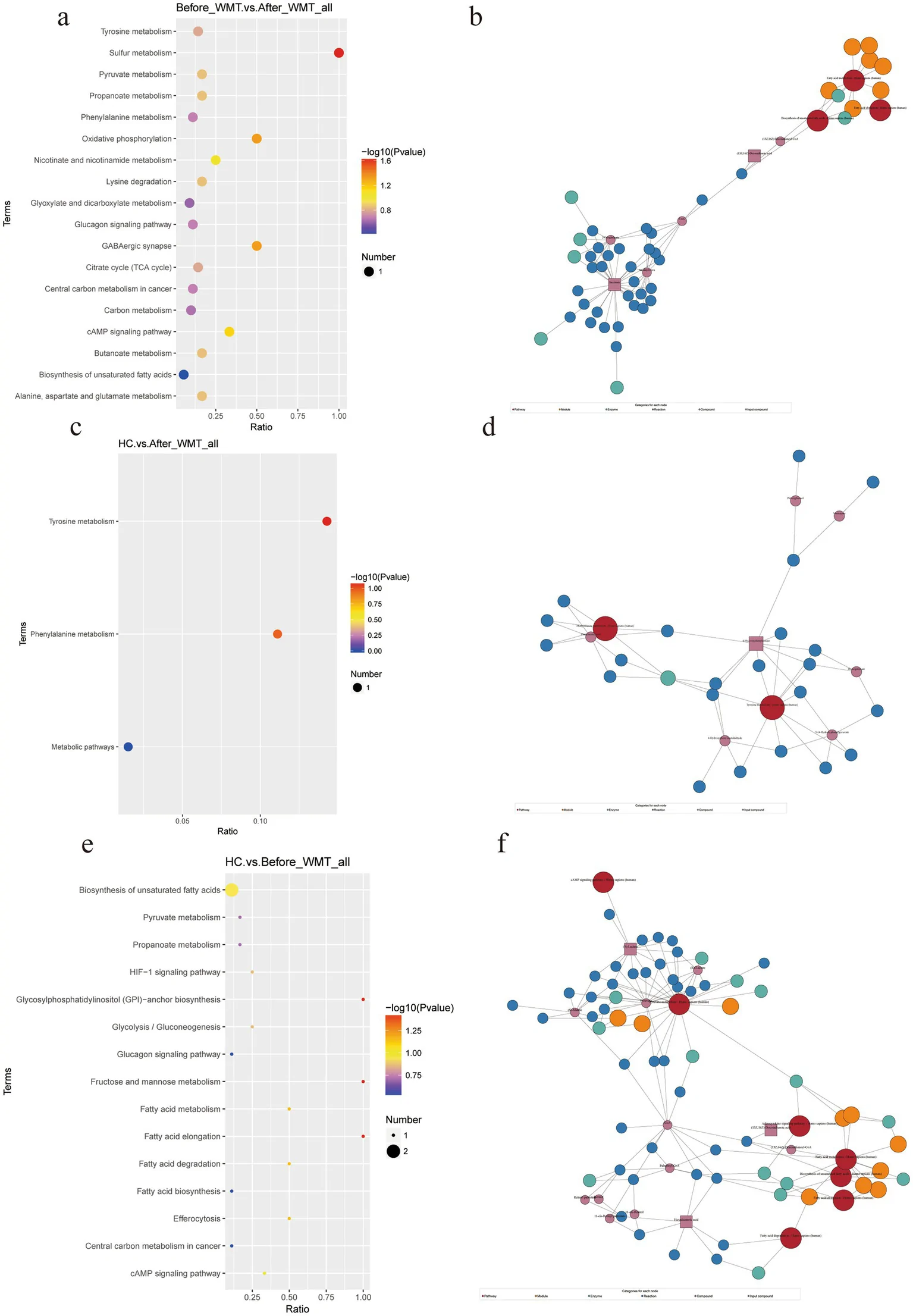
KEGG enrichment bubble plot (only showing the top 20 results) and regulatory network diagram before vs. after **(a,b)**, HC vs. after **(c,d)**, HC vs. before **(e,f)**; before (before WMT treatment); after (after WMT treatment); HC (healthy twins).

The KEGG metabolic pathway enrichment analysis revealed that there were significant differences between ASD patients before treatment and healthy controls in fructose-mannose metabolism, fatty acid elongation, and GPI anchor biosynthesis pathways. After WMT treatment, there were no significant enriched pathways between the patients and the healthy controls, and the comparison before and after treatment showed significant changes in sulfur metabolism, oxidative phosphorylation, and GABAergic synapse pathways, suggesting that WMT may be correlated with changes of the metabolic disorders related to ASD and present potential associations with energy metabolism and neural signaling pathways (Figure 7).

### 3.5 Association analysis of gut microbiota and metabolites

**Figure 8:**
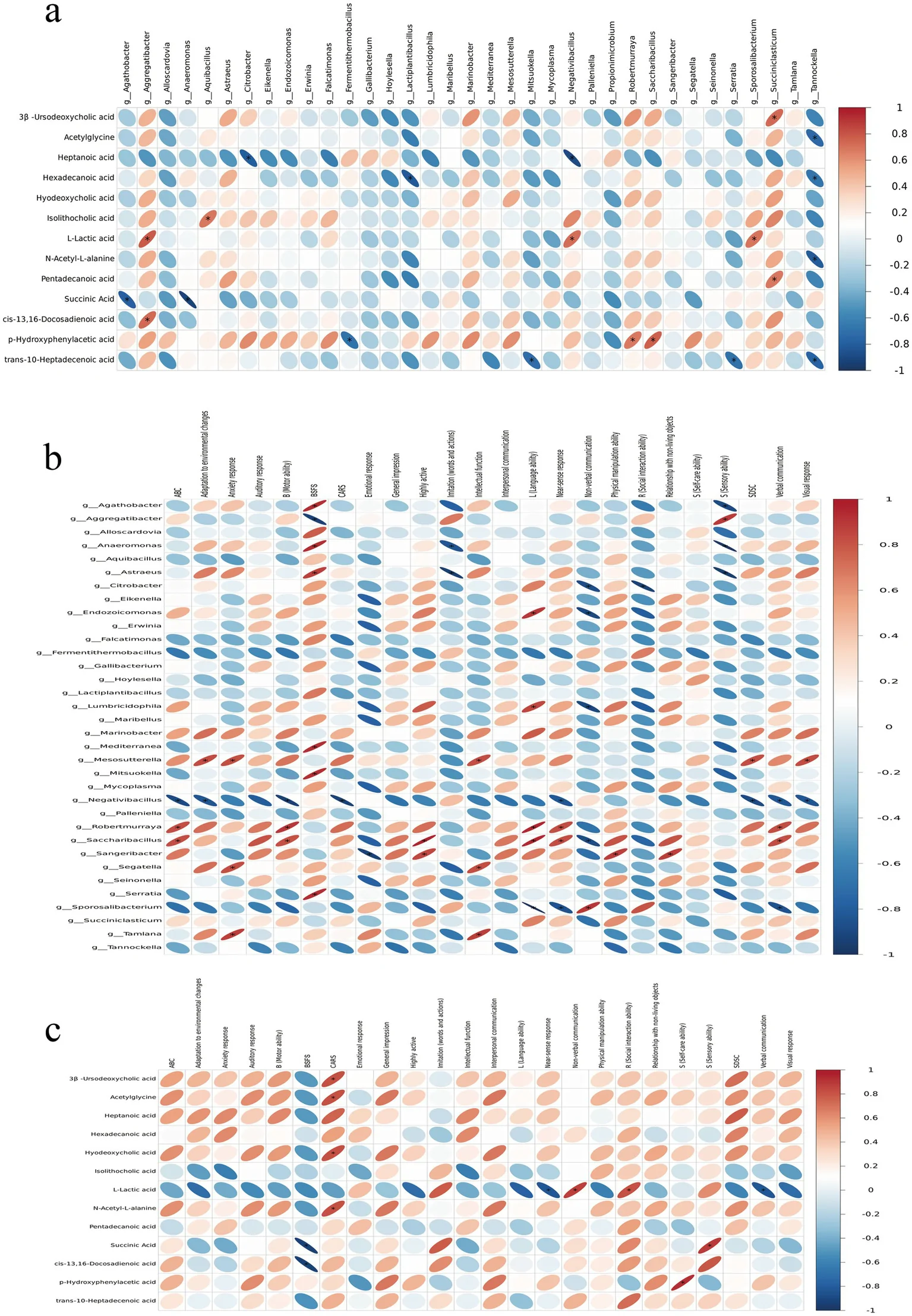
Exploratory correlation heatmaps (*n =* 3, results for observational reference only). **(a)** Correlation between differential bacterial genera and differential metabolites; **(b)** correlation between clinical scales and differential bacterial genera; **(c)** correlation between clinical scales and differential metabolites. Before, Before WMT treatment; After, After WMT treatment; HC, Healthy twins. Correlation results are not statistically conclusive due to the small sample size.

To preliminarily explore the changing trends of gut microbiota alterations after WMT and changes in host metabolic profiles, we performed Spearman rank correlation analysis on 13 differential metabolites and 35 bacterial genera at the genus level, and visualized the observed correlations via a heatmap (Figure 8a). Combined with the microbial convergent characteristics after intervention, we descriptively summarized the co-variation trends between gut microbiota and metabolites in this small cohort.

The core bacterial genera that showed convergence towards HC after intervention (such as *Agathobacter*, *Mediterranea*, *Mesosutterella*, *Hoylesella*, etc.) had associations with metabolites that were highly consistent with the inter-group changes of the metabolome. These microbes presented obvious co-variation trends with altered metabolites. *Mesosutterella*, *Agathobacter* were negatively correlated with docosadienoic acid (cis-13,16), N-acetyl-L-proline; *Agathobacter* was significantly negatively correlated with succinic acid; *Hoylesella*, *Mitsuokella,* *Mediterranea* were negatively correlated with docosadienoic acid (cis-13,16), N-acetyl-L-proline; *Paleniella*, *Maribellus* were negatively correlated with docosadienoic acid (cis-13,16) and positively correlated with N-acetyl-L-proline; *Falcrimonas* was positively correlated with docosadienoic acid (cis-13,16) and negatively correlated with N-acetyl-L-proline.

The bacterial genera that did not converge after the intervention (*Negativibacillus*, *Segatella*, *Sangeribacter*) were mainly represented in the heatmap as a negative correlation between *Segatella* docosadienoic acid (cis-13,16) and N-acetyl-L-proline; *Negativibacillus*, *Sangeribacter* were positively correlated with docosadienoic acid (cis-13,16) and N-acetyl-L-proline; *Negativibacillus* showed a significant negative correlation with heptanoic acid and a significant positive correlation with L-lactic acid. These bacterial genera did not show significant changes before and after the intervention, and were the direct cause for the specific downregulation of heptanoic acid in the After group compared to the HC group.

### 3.6 Correlation analysis of clinical scales and distinct bacterial Genuse

To preliminarily explore the co-variation trends between altered intestinal microbiota and changes in clinical phenotypes after WMT, we used Spearman rank correlation analysis to observe the correlation trends between 35 differential bacterial genera and 25 clinical scale indicators, and combined with P -values to verify the significance of the correlations, we descriptively summarized the key functional bacterial genera that were responsible for the improvement of clinical phenotypes (Figure 8b).

After WMT intervention, the core bacterial genera that showed convergence towards HC (*Mesosutterella, Agathobacter, Mediterranea*, etc.) had associations with the clinical scales that were consistent with the improvement trend of the patients’ clinical phenotypes. *Mesosutterella* was positively correlated with ABC, CARS, and negatively correlated with BSFS. It was significantly positively correlated with SDSC, adaptation to environmental changes, anxiety response, intelligence function, and visual response. *Hoylesella, Paleniella,* and *Falcrimonas* were negatively correlated with ABC, CARS, and SDSC, and positively correlated with BSFS. *Agathobacter* was negatively correlated with ABC, CARS, and SDSC, and significantly positively correlated with BSFS, and significantly negatively correlated with perception ability. *Mitsuokella* and *Mediterranea* were negatively correlated with ABC, CARS, and SDSC, and significantly positively correlated with BSFS. *Maribellus* was positively correlated with ABC and BSFS, and negatively correlated with CARS and SDSC.

The non-convergent bacterial genera (*Negativibacillus, Segatella, Sangeribacter*) did not show significant changes in abundance before and after the intervention, and there was a significant difference compared with HC. They were strongly negatively correlated with the clinical scales, presented unfavorable co-variation trends with clinical indicators, which may be related to the incomplete improvement of clinical symptoms:

*Negativibacillus* was significantly negatively correlated with ABC, CARS, SDSC, adaptation to environmental changes, B (motor ability), non-verbal communication, verbal communication, visual response, and was positively correlated with BSFS. *Segatella* was positively correlated with ABC, CARS, BSFS, and SDSC, and was significantly positively correlated with auditory response and intellectual function. *Sangeribacter* was positively correlated with ABC and CARS, was significantly positively correlated with high activity, physical operation ability, and the relationship with non-living objects, was negatively correlated with SDSC and BSFS, and was significantly negatively correlated with emotional response.

### 3.7 Correlation analysis of clinical scales and differential metabolites

To preliminarily explore the co-variation trends between altered metabolites and clinical phenotypes after WMT intervention, analysis based on Spearman rank correlation, and verified the significance of the correlations using P -values, we descriptively summarized the key metabolic targets that mediate the improvement of the clinical phenotype.

Succinic acid, docosadienoic acid (cis-13,16) were positively correlated with ABC and CARS, negatively correlated with SDSC, and significantly negatively correlated with BSFS; hyodeoxycholic acid, N-acetyl-L-proline, and acetylglycine were positively correlated with ABC and SDSC, significantly positively correlated with CARS, and negatively correlated with BSFS (Figure 8c).

## 4 Discussion

In recent years, WMT has shown promising application prospects in the clinical intervention of ASD. However, the existing clinical studies on WMT for ASD are mostly single-arm designs or unmatched case–control studies, which make it difficult to eliminate the confounding bias caused by the high heritability of ASD and cannot precisely distinguish the specific intervention effect of WMT from genetic and environmental factors that lead to microecological differences. Studies have provided evidence suggesting the functional and potential pathogenic nature of the microbiota: studies on germ-free mice colonization, ASD cohort data, and intervention studies have shown that the microbiota regulates concurrent behavioral changes (Su et al., 2024; Yu et al., 2025). Additionally, research has indicated that adjusting dietary diversity can significantly reduce the microbiota differences between children with ASD and normally developing children, and that only food selection is sufficient to cause changes in microbial composition and function (Wu et al., 2025; Mitchell et al., 2026; Zhang et al., 2025). Twins, with nearly identical genetic backgrounds and highly shared family living environments, are ideal models for analyzing the genetic and environmental effects of diseases. This study is the first to conduct a systematic exploration based on twin pairing design on the remodeling effect of WMT on the gut microbiota and fecal metabolome of children with ASD, and established a clinical phenotype–microbiota–metabolite association framework, providing high-quality genetic background-matched evidence for the mechanism of WMT in treating ASD. Compared with conventional FMT, the standardized washing and purification procedures of WMT in this study effectively remove pro-inflammatory substances and impurities, lower the risk of adverse reactions in vulnerable children with ASD, and we further combined colonoscopic TET to deliver the bacterial solution to the ileocecal region to improve the colonization efficiency of functional bacteria in the gut (Yuan et al., 2023). In addition, this study is defined as a pilot and feasibility study with only 3 twin pairs enrolled. Although the twin-paired design minimizes genetic and environmental confounding factors, the limited sample size makes it impossible to draw definitive causal conclusions. All observed associations in this work are exploratory findings and need further verification.

The results of this study show that, under the condition of consistent genetics and shared environment, the scores of CARS, ABC, and SDSC scales for children with ASD improved after WMT intervention, and the fecal quality score of BSFS tended to normal. This suggests that WMT has potential to improve the core symptoms, sleep disorders, and intestinal functions of ASD patients, which is highly consistent with the existing international research results (Feng et al., 2025). Kang et al. (2017, 2019) conducted an open-label clinical trial and first confirmed that fecal microbiota transplantation can significantly improve the gastrointestinal symptoms and behavioral symptoms of children with ASD, and the therapeutic effect can last for 2 years; subsequent multiple meta-analyses also showed that fecal microbiota transplantation intervention can significantly reduce the scores of CARS and ABC scales for ASD patients and improve core symptoms such as social skills and stereotyped behaviors (Feng et al., 2025). The sample size of this study is relatively small (3 pairs of twins), and the statistical power is limited, thus unable to provide conclusive causal evidence. However, it should be emphasized that the twin pairing design significantly enhances internal validity by minimizing the confounding effects of genetics and shared environment. According to literature reports (Isaksson et al., 2022), the sample size of ASD discordant twin studies worldwide is mostly between 10 and 20 pairs, and many proof-of-concept studies have a sample size of only 3 to 5 pairs. This study is essentially a pilot / feasibility study, aiming to provide preliminary evidence and target directions for future large-scale randomized controlled trials. The twin-paired design minimizes confounding from shared genetic background and household environment, reducing the probability that observed microbial differences are explained solely by familial genetic or shared environmental factors. However, this design cannot fully exclude non-shared environmental confounders — most notably food selectivity, restricted dietary patterns, gastrointestinal symptoms and medication use — all of which are common in ASD and are known to independently shape gut microbiota composition (Wu et al., 2025; Mitchell et al., 2026; Zhang et al., 2025). In our study, the observation that microbial and metabolic profiles shifted toward healthy twin levels following WMT, in parallel with directional changes in clinical scores, provides correlational evidence consistent with a functional role of the microbiota. Nonetheless, we cannot rule out partial secondary effects, and definitive causal inference will require prospective, diet-controlled interventional studies.

The imbalance of the intestinal microbiota structure and function is the core initiating factor for the abnormality of the “gut-brain axis” in ASD. Based on the twin-pair design, this study precisely revealed the baseline microbiota characteristics of ASD patients: Compared with healthy siblings with the same genetic background, ASD patients showed an increase in the abundance of the Firmicutes phylum and a decrease in the abundance of the Bacteroidetes phylum at the phylum level. The abundances of beneficial bacteria genera such as *Faecalibacterium* and *Bifidobacterium* were significantly insufficient, while the abundance of opportunistic pathogenic bacteria genus *Escherichia-Shigella* was abnormally enriched. This is highly consistent with the existing results from multiple global multicenter studies (Su et al., 2024; Wan et al., 2022; Jaworska et al., 2025; Tao et al., 2025; Andreo-Martínez et al., 2022; Chen et al., 2025). The *Bacteroidetes* phylum is the core dominant phylum in the human intestine, participating in polysaccharide degradation, short-chain fatty acid (SCFA) synthesis, and regulation of intestinal immune homeostasis. Its reduced abundance can lead to impaired intestinal barrier function and metabolic imbalance, thereby affecting neurodevelopment through the gut-brain axis (Wan et al., 2022); while *Faecalibacterium* is the core SCFA-producing bacteria in the intestine, and its insufficient abundance is closely related to neuroinflammation and social disorders in ASD patients (Yu et al., 2025). This study found that the WMT intervention could significantly reverse the microbial imbalance in children with ASD. After the intervention, the abundance of Bacteroidetes increased while that of Firmicutes decreased. The microbial community structure at the phylum, genus, and species levels all tended to converge towards that of healthy siblings. Among them, the genera *Agathobacter*, *Mesosutterella*, and *Hoylesella* were the core genera that converged towards that of healthy siblings after the intervention. These genera belong to the *Lachnospiraceae* and *Ruminococcaceae* families, which are important SCFAs-producing bacteria in the intestine. Previous studies have confirmed that the increase in the abundance of genera in the *Lachnospiraceae* family can significantly improve intestinal barrier function, inhibit peripheral and central nervous inflammation, and thereby improve the social behavior deficits of ASD model mice (Su et al., 2024). Functional genomic analysis further revealed that the WMT intervention could significantly down-regulate the abnormal activated genetic information processing, environmental information processing, and neurodegenerative disease-related pathways in children with ASD, and repair the core modules related to oxidative stress, methylation function, and energy metabolism. This is highly consistent with the pathological and physiological characteristics of ASD. Previous studies have confirmed (Dan et al., 2020; Sharon et al., 2019; van de Wouw et al., 2024; Frye et al., 2024) that children with ASD have significantly enhanced oxidative stress, impaired methylation cycle, and abnormal mitochondrial function. The intestinal microbiota can regulate the host’s oxidative stress level and mitochondrial function through metabolites, thereby affecting neuronal development and synaptic transmission. The results of this study provide valuable functional genomic evidence that can be used to understand the pathological pathways related to WMT and ASD.

In terms of donor selection, we adopted strictly screened healthy adult donors in this pilot study. Current clinical data demonstrate that short-term WMT using qualified adult microbiota is safe for children with ASD (Feng et al., 2025). Meanwhile, we acknowledge the potential theoretical concern that adult-derived microbiota may affect the development of children’s gut microecology. Due to objective limitations such as the lack of sufficient age-matched pediatric donors, this study did not set a pediatric donor control group. In future large-scale trials, we will add age-matched pediatric donors to carry out comparative research, so as to optimize the donor selection scheme for pediatric ASD population.

The metabolites produced by the gut microbiota are the core mediators of the “gut-brain axis” signaling communication. The results of this metabolomics study showed that WMT intervention could specifically reshape the lipid metabolic profile of children with ASD. After the intervention, the differentially metabolized substances between the ASD patients and their healthy siblings decreased from 8 to 2. The core downregulated metabolites included succinic acid, deoxycholic acid, and docosadienoic acid, etc. The core regulatory pathway was the biosynthesis of unsaturated fatty acids and fatty acid metabolism. Docosadienoic acid, as an omega-6 series polyunsaturated fatty acid, is an important precursor for the synthesis of neural cell membranes. Its downregulation may improve the physiological state of neural cells, providing a metabolic basis for the alleviation of ASD symptoms. It is worth noting that after WMT intervention, there were still differences in the aromatic amino acid metabolic pathways between the ASD patients and their healthy siblings. Aromatic amino acids are precursors of monoamine neurotransmitters such as dopamine and 5-hydroxytryptamine. Their abnormal metabolism is closely related to the social disorders and emotional abnormalities of ASD, suggesting that a single course of WMT intervention cannot completely reverse the amino acid metabolic abnormalities in ASD patients. This also provides an important direction for optimizing the WMT intervention plan and combining amino acid targeted supplementation. The functional pathway annotations derived from KEGG, EggNOG, CAZy, and other databases are based on computational predictions from metagenomic sequencing data. These assignments should be interpreted as hypothesis-generating rather than definitive evidence of metabolic activity. The actual expression and activity of these pathways require confirmation through transcriptomic, proteomic, or metabolomic experimental validation.

This study, through multi-omics association analysis, for the first time constructed an association of “clinical phenotype - microbiota - metabolites” in a model with matched genetic background. The results showed that the core bacterial genera such as *Agathobacter*, *Mesosutterella*, and *Hoylesella*, which tended to converge towards healthy siblings after WMT intervention, were significantly negatively correlated with differential metabolites such as docosadienoic acid and N-acetyl-L-proline, and were also significantly negatively correlated with CARS and ABC scale scores. This suggests that these bacterial genera can mediate the improvement of core symptoms of ASD by regulating lipid and amino acid metabolic products. While the bacterial genera such as *Segatella*, *Negativibacillus*, and *Sangeribacter* that did not tend to converge towards healthy siblings after the intervention were strongly negatively correlated with clinical scale scores, and were the key bottleneck restricting the complete improvement of ASD clinical symptoms. This result initially explored the core functional bacterial genera and metabolic targets for improving the symptoms of ASD through WMT, providing a biological reference basis for the subsequent development of precise microecological preparations targeting ASD.

## 5 Limitations and prospects

First, the small sample size (3 twin pairs) greatly limits statistical power, and all observed associations are exploratory rather than causal. Second, this is a single-center, non-randomized observational pilot study, and the relevant mechanism evidence is only correlational. Third, we used healthy adult donors instead of age-matched pediatric donors, which is an objective limitation of this study. Fourth, healthy co-twins were sampled only at baseline without longitudinal follow-up, which limits the ability to exclude time-dependent microbiome and metabolome fluctuations unrelated to WMT. Future studies should include serial sampling of both ASD and healthy co-twins across multiple time points to better distinguish intervention-specific effects from natural temporal variation. This pilot study did not include dedicated microbial engraftment assessment through longitudinal strain-level tracking. Future studies will incorporate temporal microbiome profiling to formally evaluate donor microbiota colonization and persistence. Potential confounders including diet, concurrent behavioral therapies, and medication history were monitored but not formally controlled through randomization or stratification, which is a limitation of this observational pilot design. Future randomized controlled trials should standardize and formally control these variables. The retrospective trial registration was due to administrative procedural delays rather than protocol modifications or selective reporting.

Despite these limitations, the twin-paired design provides high internal validity and strengthens causal inference regarding WMT effects in ASD. Future research should combine organoids and animal models to deeply analyze the molecular mechanism of the “microbiota - metabolites - intestine - brain axis” in regulating ASD. Conduct large-scale, multi-center, randomized, double-blind, placebo-controlled trials to verify the efficacy and safety of WMT. Compare the therapeutic effects of adult donors and pediatric donors, and optimize the donor selection strategy. Evaluate the long-term efficacy of multiple-course WMT and determine the optimal treatment plan.

## 6 Conclusion

In this small twin pilot cohort with matched genetic and environmental backgrounds, WMT intervention was associated with measurable remodeling of gut microbial structure and function, as well as partial normalization of fecal metabolic profiles in children with ASD. These microbial and metabolic changes shifted directionally toward the profile of healthy twins and involved pathways relevant to the microbiota–gut–brain axis. Numerical improvements in clinical symptom scores were observed but did not reach statistical significance. These should be interpreted as preliminary exploratory trends and do not constitute evidence of clinical efficacy. Several key microbial taxa and metabolic pathways showing phenotypic co-variation were identified, providing candidate targets for subsequent mechanistic research. This pilot work provides preliminary feasibility data and mechanistic clues supporting WMT as a potential microbiome-targeted intervention for ASD. Large-sample, multi-center randomized controlled trials are urgently needed to further verify the efficacy, safety and underlying mechanisms of WMT.

## Data Availability

The raw sequencing data are publicly available in the NCBI SRA database under BioProject accession PRJNA1458179: http://www.ncbi.nlm.nih.gov/bioproject/1458179. The raw targeted metabolomics data can be found in Supplementary Table 1. Individual anonymized clinical data of all enrolled twin participants (including all baseline and post-intervention scale scores) are presented in Supplementary Table 2. All clinical, metagenomic and metabolomics data can be obtained by reasonable request to the corresponding authors.
